# Gender Differences in Clinical Outcomes among HIV-Positive Individuals on Antiretroviral Therapy in Canada: A Multisite Cohort Study

**DOI:** 10.1371/journal.pone.0083649

**Published:** 2013-12-31

**Authors:** Angela Cescon, Sophie Patterson, Keith Chan, Alexis K. Palmer, Shari Margolese, Ann N. Burchell, Curtis Cooper, Marina B. Klein, Nima Machouf, Julio S. G. Montaner, Chris Tsoukas, Robert S. Hogg, Janet M. Raboud, Mona R. Loutfy, CANOC Collaboration

**Affiliations:** 1 British Columbia Centre for Excellence in HIV/AIDS, Vancouver, Canada; 2 Faculty of Health Sciences, Simon Fraser University, Burnaby, Canada; 3 Canadian Observational Cohort (CANOC) collaboration, Toronto, Canada; 4 Ontario HIV Treatment Network, Toronto, Canada; 5 University of Ottawa, The Ottawa Hospital Research Institute, Ottawa, Canada; 6 Department of Medicine, McGill University, Montreal, Canada; 7 Montreal Chest Institute, McGill University Health Centre, Montreal, Canada; 8 Clinique médicale l’Actuel, Montreal, Canada; 9 Division of AIDS, Department of Medicine, University of British Columbia, Vancouver, Canada; 10 Dalla Lana School of Public Health, University of Toronto, Toronto, Canada; 11 Division of Infectious Diseases, University Health Network, Toronto, Canada; 12 Women’s College Research Institute, Women’s College Hospital, Toronto, Canada; 13 Maple Leaf Medical Clinic, Toronto, Canada; 14 Department of Medicine, University of Toronto, Toronto, Canada; Vanderbilt University, United States of America

## Abstract

**Background:**

Cohort data examining differences by gender in clinical responses to combination antiretroviral therapy (ART) remain inconsistent and have yet to be explored in a multi-province Canadian setting. This study investigates gender differences by injection drug use (IDU) history in virologic responses to ART and mortality.

**Methods:**

Data from the Canadian Observational Cohort (CANOC) collaboration, a multisite cohort study of HIV-positive individuals initiating ART after January 1, 2000, were included. This analysis was restricted to participants with a follow-up HIV-RNA plasma viral load measure and known IDU history. Weibull hazard regression evaluated time to virologic suppression (2 consecutive measures <50 copies/mL), rebound (>1000 copies/mL after suppression), and all-cause mortality. Sensitivity analyses explored the impact of presumed ART use in pregnancy on virologic outcomes.

**Results:**

At baseline, women (1120 of 5442 participants) were younger (median 36 vs. 41 years) and more frequently reported IDU history (43.5% vs. 28.8%) (both p<0.001). Irrespective of IDU history, in adjusted multivariable analyses women were significantly less likely to virologically suppress after ART initiation and were at increased risk of viral load rebound. In adjusted time to death analysis, no differences by gender were noted. After adjusting for presumed ART use in pregnancy, observed gender differences in time to virologic suppression for non-IDU, and time to virologic rebound for IDU, became insignificant.

**Conclusions:**

HIV-positive women in CANOC are at heightened risk for poor clinical outcomes. Further understanding of the intersections between gender and other factors augmenting risk is needed to maximize the benefits of ART.

## Introduction

Despite substantial reductions in morbidity and mortality since the introduction of combination antiretroviral therapy (ART), HIV and AIDS continue to be public health concerns in Canada [1]. Mirroring global trends, the profile of the HIV epidemic in Canada has changed, with a notable incidence escalation among women over the past 15 years [1], [2]. In 2011 an estimated 16,600 women were living with HIV, compared to 14,740 in 2008 [1]. In terms of distribution trends, the proportion of positive HIV tests among women has steadily increased, with women accounting for 24% of adult positive tests in 2011; double the 12% proportion observed from 1985–1998 [1].

Women have unique experiences of HIV infection and ART, which need to be further explored. Published studies largely agree that following seroconversion women demonstrate higher CD4 T-cell counts and lower HIV-RNA viral load measures than men [3]–[7]. However, cohort data examining gender differences in response to therapy and disease progression remain contradictory [8], and are likely influenced by context, setting, and other social determinants that augment access to ART and associated support services [9].

Investigating clinical outcomes by gender allows for improved understanding of trends in a region’s epidemic, to inform policy and practice and to facilitate the creation of effective gender-specific programming where most needed. In Canada, gender differences in clinical responses to ART have yet to be explored using data collated from multiple provinces. This study investigates gender differences by injection drug use (IDU) history in virologic responses to ART and mortality among persons living with HIV across Canada.

## Methods

### Data source

The Canadian Observational Cohort (CANOC) collaboration is a multisite cohort study of HIV-positive individuals initiating combination ART after January 1, 2000. Currently, eight cohorts contribute data to CANOC, representing the country’s three largest provinces (Ontario, British Columbia, and Quebec). CANOC is the largest collaborative cohort in Canada focused on gaining a better understanding of HIV therapeutics after they have been released on the market, capturing a third of the estimated 24,000 people on HIV treatment in the represented provinces [10].

Data collection and extraction are performed locally at the data centres of the participating sites, and is then pooled and analyzed at the coordinating centre in Vancouver, British Columbia. All participating cohorts have received ethical approval from their institutional boards to contribute data to this collaboration. Further details on the collaborating cohorts and general CANOC structure have been published previously [10]. The last date of follow-up in the cohort for the current analysis was September 30, 2011.

### Inclusion criteria

For inclusion in CANOC, patients must be at least 18 years old, have documented HIV infection, reside in Canada, have initiated ART with at least three individual agents naively (i.e., no prior antiretroviral experience) on or after January 1, 2000, and have baseline (within six months of ART initiation) CD4 cell count and viral load testing results. To be included in this analysis, participants required at least one follow-up viral load measurement and a non-missing IDU history.

### Outcomes and statistical methods

Primary outcomes of interest included (1) time from ART initiation to virologic suppression, defined as having two consecutive viral load measures (at least 30 days apart) below 50 copies/mL; (2) time to virologic rebound, defined as a measure above 1000 copies/mL after suppression; and (3) time from ART initiation to death (all-cause). Kaplan-Meier methods were used to compare time to events by gender and IDU history status. Weibull hazard regression was used to model all outcomes, as proportional hazards assumptions were not met. Mortality data were obtained through either physician reporting or linkage to provincial vital statistics registries. For all outcomes, the primary covariate of interest was gender by IDU history. IDU history was defined as a documented HIV risk factor of injection drug use, ascertained from a combination of surveys, physician interviews, and medical record data.

Demographic and clinical characteristics of interest included age, province, risk factors for HIV infection, baseline AIDS-defining illnesses, hepatitis C (HCV) co-infection, baseline CD4 cell count and viral load, rate of viral load monitoring (number of tests per year), composition of initial ART regimen, and year of ART initiation. Participants were classified as HCV co-infected if ever identified as HCV-positive through physician reports, antibody test results, or PCR test results. Demographic and clinical characteristics were summarized using frequencies and percentages for categorical variables and medians with interquartile range (IQR) for continuous variables. Categorical characteristics were compared between men and women using the Pearson χ^2^ test or the Fisher’s exact test. Continuous variables were compared using the Wilcoxon rank-sum test.

To explore the impact of ART use solely for prevention of vertical HIV transmission on virologic rebound and suppression outcomes, sensitivity analyses were performed based on an adapted version of an algorithm used previously to identify presumed pregnancies in HIV cohort studies lacking explicit pregnancy data [11]. Algorithm criteria included: female; baseline age <46 years; initiation of an ART regimen containing lamivudine/zidovudine and one of nevirapine, nelfinavir, lopinavir, or saquinavir; and, remaining on the regimen for more than 30 days but less than one year. Women identified as taking ART for prevention of vertical HIV transmission via this algorithm were excluded from these sub-analyses.

In all analyses the “intent to treat” approach was used, whereby included ART data were based on regimens prescribed at baseline (i.e., first therapy). Participants without outcomes of interest during follow-up were censored as of the date of their last viral load (virologic outcomes), or date of last contact or end of study period, whichever came first (mortality analysis). Statistical analyses were performed using SAS software version 9.1.3 (SAS Institute, Cary, North Carolina).

## Results

### Study population

A total of 5442 participants met the study inclusion criteria, 20.6% (n = 1120) women. Reasons for exclusion included missing data on gender (n = 40) and IDU history (n = 2376). At baseline, the median age of all participants was 40 years (IQR 34–46); CD4 cell count 200 cells/mm^3^ (IQR 110–290); and viral load 4.9 log_10_ copies/mL (IQR 4.4–5.0). 42.0% of participants (n = 2284) were from British Columbia, 30.2% (n = 1643) from Ontario, and 27.8% (n = 1515) from Quebec. In the total median follow-up time of 49 months (IQR 25–83), 462 deaths were reported and 548 participants were lost to follow-up (defined as no contact for ≥18 months).

At pre-ART baseline women were younger than male counterparts (median 36 years vs. 41), had lower viral load measurements (median 4.6 log_10_ copies/mL vs. 4.9), and a higher proportion reported IDU history (43.5% vs. 28.8%) (all p<0.001) (**table 1**). Viral load monitoring rate differed significantly by gender, with women receiving fewer tests per year (p<0.001). Initial ART regimens also differed significantly between men and women, with male participants more frequently prescribed NNRTI or boosted PI-based regimens (p<0.001). The prevalence of HCV co-infection was significantly greater among female participants (p<0.001). No significant differences by gender were observed in baseline CD4 cell count (p = 0.787) or total follow-up time (p = 0.507).

**Table 1:** Baseline demographic and clinical characteristics by gender (n = 5442).

Variable	Total (n = 5442)		Gender				p-value
			Female (n = 1120)		Male (n = 4322)		
Age (years)	40	(34–46)	36	(30–43)	41	(35–47)	<0.001
**Province**							
*British Columbia*	2284	(42.0)	517	(46.2)	1767	(40.9)	<0.001
*Ontario*	1643	(30.2)	336	(30.0)	1307	(30.2)	
*Quebec*	1515	(27.8)	267	(23.8)	1248	(28.9)	
**Heterosexual HIV risk** ¶							
*No*	3344	(62.5)	386	(35.1)	2958	(69.6)	<0.001
*Yes*	2003	(37.5)	146	(64.9)	1289	(30.4)	
**History of IDU**							
*No*	3711	(68.2)	633	(56.5)	3078	(71.2)	<0.001
*Yes*	1731	(31.8)	487	(43.5)	1244	(28.8)	
**Baseline ADI^+^**							
*No*	4508	(84.0)	961	(86.8)	3547	(83.2)	0.004
*Yes*	860	(16.0)	146	(13.2)	714	(16.8)	
**Hepatitis C co-infection^?^**							
*No*	3562	(68.1)	596	(55.0)	2966	(71.5)	<0.001
*Yes*	1668	(31.9)	488	(45.0)	1180	(28.5)	
CD4 count (cells/mm^3^)	200	(110–290)	200	(118–284)	203	(109–298)	0.787
**Viral load (log_10_ copies/mL)**	4.9	(4.4–5.0)	4.6	(4.0–5.0)	4.9	(4.4–5.0)	<0.001
VL testing rate (tests/year)^*^							
*<3*	1461	(27.0)	380	(34.1)	1081	(25.2)	<0.001
*3*–*4*	1489	(27.5)	325	(29.1)	1164	(27.1)	
*5*–*6*	1732	(32.0)	309	(27.7)	1423	(33.1)	
*>6*	730	(13.5)	101	(9.1)	629	(14.6)	
Follow-up time (months)	49	(25–83)	49	(25–83)	48	(25–82)	0.507
Year of ART initiation	2006	(‘03– ‘08)	2005	(‘03– ‘08)	2006	(‘03– ‘08)	0.071
**Third ARV class** §							
*NNRTI*	2425	(44.6)	477	(42.6)	1948	(45.1)	<0.001
*Single PI*	713	(13.1)	200	(17.9)	513	(11.9)	
*Boosted PI*	2182	(40.1)	414	(37.0)	1768	(40.9)	
*NRTI*	122	(2.2)	29	(2.6)	93	(2.2)	
**Third ARV agent** §							
*Nevirapine*	596	(11.0)	147	(13.1)	449	(10.4)	<0.001
*Efavirenz*	1823	(33.5)	329	(29.4)	1494	(34.6)	
*Lopinavir*	979	(18.0)	201	(17.9)	778	(18.0)	
*Atazanavir*	1278	(23.5)	258	(23.0)	1020	(23.6)	
*Other*	766	(14.1)	185	(16.5)	581	(13.4)	

Results presented as median (IQR) or frequency (%).

Note: IDU  =  injection drug use; ADI  =  AIDS-defining illness; VL  =  viral load; ARV  =  antiretroviral; NRTI  =  nucleoside reverse transcriptase inhibitor; NNRTI  =  non-nucleoside reverse transcriptase inhibitor; PI  =  protease inhibitor.

n  =  5347; ^+^n  =  5368; ^?^n  =  5230; ^*^n  =  5412.

Alongside two NRTIs.

### Clinical outcomes among IDU

**Figure 1:**
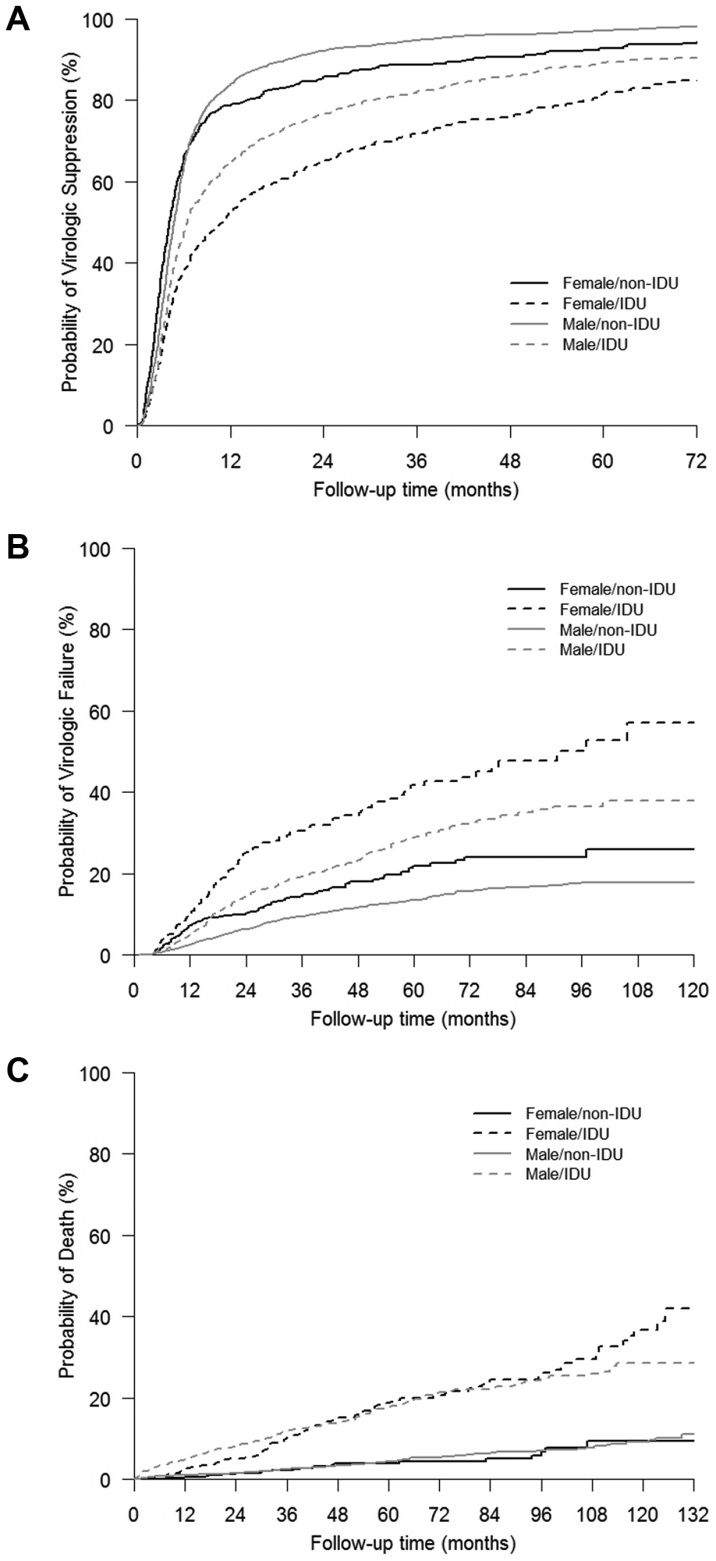
Time to virologic suppression (A), virologic rebound (B), and death (C) by gender and IDU history.

Using Kaplan-Meier methods, the estimated probability of virologic suppression among individuals with IDU history was 0.38 (95% CI = 0.34–0.42) and 0.52 (95% CI = 0.48–0.57) for women and 0.47 (95% CI = 0.44–0.50) and 0.65 (95% CI = 0.62–0.67) for men, at 6 and 12 months post-ART initiation, respectively. For virologic rebound, probabilities were 0.04 (95% CI = 0.02–0.06) and 0.10 (95% CI = 0.07–0.13) for women and 0.01 (95% CI = 0.00–0.01) and 0.05 (95% CI = 0.04–0.06) for men, at 6 and 12 months after virologic suppression, respectively. Among women, mortality rates at 12 and 24 months after ART initiation were 0.03 (95% CI = 0.01–0.04) and 0.05 (95% CI = 0.03–0.07). For men, mortality rates at the same time points were 0.05 (95% CI = 0.04–0.06) and 0.08 (95% CI = 0.06–0.10) (**figure 1**).

Unadjusted results for virologic suppression, virologic rebound, and mortality among individuals with IDU history are available in **table 2**. In adjusted multivariable analysis, women were significantly less likely to achieve virologic suppression compared to men (AHR = 0.82, 95% CI = 0.72–0.93, p = 0.002) and were at increased risk of virologic rebound (AHR = 1.31, 95% CI = 1.03–1.66, p = 0.026) (**table 3**). There was no significant difference by gender in time to death after ART initiation (AHR = 1.21, 95% CI = 0.94–1.55, p = 0.143) (**table 3**)**.**

**Table 2:** Unadjusted analyses exploring gender differences in clinical outcomes among individuals with IDU history.

OUTCOME	Virologic Suppression (n = 1703)		Virologic Rebound (n = 1336)		Mortality (n = 1731)		
Variable	Unadjusted HR (95% CI)	p-value	Unadjusted HR (95% CI)	p-value	Unadjusted HR (95% CI)	p-value	
**Gender (female vs. male)**	0.72 (0.64,0.81)	<0.001	1.68 (1.34,2.11)	<0.001	1.07 (0.84,1.36)	0.581	
**Age (per decade)**	1.32 (1.24,1.40)	<0.001	0.58 (0.51,0.66)	<0.001	1.37 (1.21,1.56)	<0.001	
**Province**							
*British Columbia*	1.00		1.00		1.00		
*Ontario*	1.12 (0.94,1.33)	0.193	0.51 (0.33,0.80)	0.003	0.33 (0.19,0.59)	<0.001	
*Quebec*	1.20 (1.00,1.44)	0.051	0.67 (0.45,0.99)	0.044	0.73 (0.48,1.11)	0.147	
**Heterosexual HIV risk**	1.00 (0.90,1.11)	0.990	1.00 (0.80,1.24)	0.986	0.34 (0.26,0.44)	<0.001	
**Baseline ADI**	1.04 (0.90,1.21)	0.563	0.73 (0.54,0.99)	0.042	1.19 (0.89,1.57)	0.234	
**Hepatitis C co-infection**	0.64 (0.56,0.74)	<0.001	2.02 (1.41,2.90)	<0.001	2.82 (1.75,4.54)	<0.001	
**Third ARV class**							
*Triple NRTI*	1.00		1.00		1.00		
*NNRTI*	1.09 (0.71,1.68)	0.698	0.91 (0.45,1.85)	0.791	7.40 (1.04,52.85)	0.046	
*Single PI*	0.77 (0.49,1.22)	0.262	1.10 (0.52,2.32)	0.812	4.72 (0.64,34.77)	0.127	
*Boosted PI*	1.12 (0.72,1.73)	0.619	0.77 (0.37,1.57)	0.466	7.09 (0.99,50.74)	0.051	
**Third ARV agent**							
*Nevirapine*	1.00		1.00		1.00		
*Efavirenz*	1.65 (1.39,1.96)	<0.001	0.52 (0.38,0.72)	<0.001	1.21 (0.88,1.67)	0.242	
*Lopinavir*	1.28 (1.06,1.54)	0.011	0.48 (0.34,0.69)	<0.001	1.23 (0.87,1.73)	0.236	
*Atazanavir*	1.57 (1.32,1.87)	<0.001	0.64 (0.46,0.88)	0.006	0.89 (0.62,1.27)	0.512	
*Other*	0.87 (0.71,1.08)	0.211	1.03 (0.75,1.41)	0.869	0.72 (0.47,1.08)	0.114	
**Baseline CD4 count (/100 cells)**	0.99 (0.96,1.02)	0.386	1.11 (1.03,1.19)	0.004	0.86 (0.79,0.94)	<0.001	
**Baseline viral load (/log_10_)**	0.78 (0.72,0.84)	<0.001	1.13 (0.94,1.36)	0.207	1.18 (0.96,1.45)	0.126	
**VL testing rate (tests/year)**							
*<3*	1.00		1.00				
*3*–*4*	1.95 (1.68,2.26)	<0.001	1.33 (0.98,1.79)	0.066			
*5*–*6*	2.04 (1.77,2.35)	<0.001	1.04 (0.77,1.40)	0.795			
*>6*	2.36 (1.96,2.83)	<0.001	1.13 (0.76,1.68)	0.554			
**Year of ART initiation**	1.08 (1.06,1.10)	<0.001	0.89 (0.86,0.93)	<0.001	1.02 (0.98,1.06)	0.333	

Note: HR  =  hazard ratio; CI  =  confidence interval; IDU  =  injection drug use; ADI  =  AIDS-defining illness; VL  =  viral load; ARV  =  antiretroviral; NRTI  =  nucleoside reverse transcriptase inhibitor; NNRTI  =  non-nucleoside reverse transcriptase inhibitor; PI  =  protease inhibitor.

**Table 3:** Adjusted multivariable analyses exploring gender differences by IDU history in clinical outcomes, with and without ART use in pregnancy exclusion.

OUTCOME	Virologic Suppression*		Virologic Rebound+		Mortality#	
Variable	Adjusted HR (95% CI)	p-value	Adjusted HR (95% CI)	p-value	Adjusted HR (95% CI)	p-value
IDU:						
Gender (female vs. male)	0.82 (0.72,0.93)	0.002	1.31 (1.03,1.66)	0.026	1.21 (0.94,1.55)	0.143
Gender (female vs. male), *excluding presumed pregnancies*	0.87 (0.76,0.99)	0.032	1.15 (0.89,1.48)	0.288	-	-
Non-IDU:						
Gender (female vs. male)	0.87 (0.79,0.96)	0.004	1.55 (1.19,2.02)	0.001	1.18 (0.75,1.85)	0.476
Gender (female vs. male), *excluding presumed pregnancies*	0.94 (0.85,1.04)	0.254	1.43 (1.07,1.91)	0.016	-	-

Note: HR  =  hazard ratio; CI  =  confidence interval; IDU  =  injection drug use.

Adjusted for age, province, VL testing rate, year therapy started, 3^rd^ ARV, baseline viral load, baseline CD4 count.

Adjusted for age, province, VL testing rate, year therapy started, 3^rd^ ARV, baseline viral load, baseline CD4 count.

^#^ Adjusted for age, province, year therapy started, 3^rd^ ARV class, baseline viral load, baseline CD4 count.

### Clinical outcomes among non-IDU

Using Kaplan-Meier methods, the estimated probability of virologic suppression among individuals without IDU history was 0.64 (95% CI = 0.60–0.68) and 0.79 (95% CI = 0.75–0.82) for women and 0.63 (95% CI = 0.61–0.65) and 0.84 (95% CI = 0.82–0.85) for men, at 6 and 12 months post-ART initiation, respectively. For virologic rebound, probabilities were 0.02 (95% CI = 0.01–0.03) and 0.07 (95% CI = 0.05–0.10) for women and 0.01 (95% CI = 0.00–0.01) and 0.03 (95% CI = 0.02–0.03) for men, at 6 and 12 months after virologic suppression, respectively. Among women, mortality rates at 12 and 24 months after ART initiation were 0.01 (95% CI = 0.00–0.01) and 0.02 (95% CI = 0.01–0.03). For men, mortality rates at the same time points were 0.01 (95% CI = 0.01–0.01) and 0.02 (95% CI = 0.01–0.02) (**figure 1**).

Unadjusted results for virologic suppression, virologic rebound, and mortality among non-IDU are available in **table 4**. In adjusted multivariable analysis, women were significantly less likely to achieve virologic suppression compared to men (AHR = 0.87, 95% CI = 0.79–0.96, p = 0.004) and were at increased risk of virologic rebound (AHR** = **1.55, 95% CI = 1.19–2.02, p = 0.001) (**table 3**). There was no significant difference by gender identified in time to death from commencement of ART (AHR = 1.18, 95% CI 0.75–1.85, p = 0.476) (**table 3**).

**Table 4:** Unadjusted analyses exploring gender differences in clinical outcomes among individuals without IDU history.

OUTCOME	Virologic Suppression (n = 3654)		Virologic Rebound (n = 3236)		Mortality (n = 3711)	
Variable	Unadjusted HR (95% CI)	p-value	Unadjusted HR (95% CI)	p-value	Unadjusted HR (95% CI)	p-value
**Gender (female vs. male)**	0.80 (0.73,0.88)	<0.001	1.64 (1.28,2.10)	<0.001	0.89 (0.57,1.38)	0.595
**Age (per decade)**	1.13 (1.09,1.17)	<0.001	0.73 (0.65,0.82)	<0.001	1.77 (1.54,2.04)	<0.001
**Province**						
*British Columbia*	1.00	0.349	1.00		1.00	
*Ontario*	0.96 (0.88,1.05)	0.084	0.89 (0.70,1.13)	0.343	0.47 (0.33,0.69)	<0.001
*Quebec*	0.92 (0.85,1.01)	0.349	0.76 (0.58,0.98)	0.035	0.48 (0.32,0.71)	<0.001
**Heterosexual HIV risk**	0.97 (0.90,1.04)	0.348	1.30 (1.05,1.60)	0.014	0.82 (0.58,1.15)	0.255
**Baseline ADI**	1.01 (0.92,1.11)	0.805	0.86 (0.65,1.14)	0.298	2.08 (1.47,2.95)	<0.001
**Hepatitis C co-infection**	0.88 (0.76,1.01)	0.076	1.96 (1.43,2.70)	<0.001	2.43 (1.54,3.83)	<0.001
**Third ARV class**						
*Triple NRTI*	1.00	<0.001	1.00	0.007	1.00	0.669
*NNRTI*	1.74 (1.38,2.19)	0.528	0.54 (0.34,0.85)	0.102	1.25 (0.45,3.44)	0.620
*Single PI*	1.08 (0.85,1.38)	<0.001	0.66 (0.40,1.09)	0.016	1.31 (0.45,3.80)	0.225
*Boosted PI*	1.59 (1.26,2.00)	<0.001	0.57 (0.36,0.90)	0.007	1.87 (0.68,5.13)	0.669
**Third ARV agent**						
*Nevirapine*	1.00		1.00		1.00	
*Efavirenz*	1.45 (1.28,1.63)	<0.001	0.52 (0.38,0.72)	<0.001	0.69 (0.41,1.16)	0.162
*Lopinavir*	1.08 (0.95,1.24)	0.231	0.69 (0.49,0.97)	0.033	1.18 (0.70,1.96)	0.535
*Atazanavir*	1.43 (1.26,1.63)	<0.001	0.45 (0.31,0.67)	<0.001	0.97 (0.55,1.70)	0.919
*Other*	0.71 (0.61,0.81)	<0.001	1.15 (0.84,1.56)	0.391	0.90 (0.53,1.53)	0.690
**Baseline CD4 count (/100 cells)**	0.99 (0.97,1.01)	0.394	1.19 (1.12,1.26)	<0.001	0.84 (0.75,0.95)	0.006
**Baseline viral load (/log_10_)**	0.95 (0.90,0.99)	0.026	0.97 (0.84,1.12)	0.645	1.40 (1.04,1.88)	0.028
**VL testing rate (tests/year)**						
*<3*	1.00		1.00			
*3*–*4*	1.85 (1.68,2.04)	<0.001	0.65 (0.49,0.87)	0.004		
*5*–*6*	1.83 (1.66,2.01)	<0.001	0.97 (0.74,1.28)	0.836		
*>6*	1.59 (1.41,1.81)	<0.001	1.82 (1.31,2.53)	<0.001		
**Year of ART initiation**	1.08 (1.07,1.10)	<0.001	0.88 (0.84,0.92)	<0.001	0.99 (0.93,1.05)	0.717

Note: HR  =  hazard ratio; CI  =  confidence interval; IDU  =  injection drug use; ADI  =  AIDS-defining illness; VL  =  viral load; ARV  =  antiretroviral; NRTI  =  nucleoside reverse transcriptase inhibitor; NNRTI  =  non-nucleoside reverse transcriptase inhibitor; PI  =  protease inhibitor.

### Sensitivity analyses

Via the algorithm described previously, 154 women were identified as potentially taking ART for prevention of vertical HIV transmission. When excluding these women, the difference in time to virologic suppression between men and women with IDU history remained statistically significant (AHR = 0.87, 95% CI = 0.76–0.99, p = 0.032), however the difference by gender in time to virologic rebound was no longer apparent (AHR = 1.15, 95% CI = 0.89–1.48, p = 0.288) (**table 3**). For participants without IDU history, the difference by gender in time to virologic suppression lost statistical significance (AHR = 0.94, 95% CI = 0.85–1.04, p = 0.254), however the significant difference in time to virologic rebound remained (AHR = 1.43, 95% CI = 1.07–1.91, p = 0.016) (**table 3**).

## Discussion

This study is the first to compare clinical responses to ART and survival by gender among HIV-positive individuals from multiple provinces in Canada, thus contributing a number of novel findings on the impacts of modern ART for this region. This multisite cohort analysis demonstrates that women and men differ significantly in all baseline demographic and clinical factors examined except for year of ART initiation and baseline CD4 cell count, highlighting the substantial variations by gender within our cohort. Irrespective of IDU history, women demonstrated poorer responses to ART in terms of virologic suppression and virologic rebound compared with male participants; however, significant differences by gender in survival were not observed.

Female participants, most notably those with a history of IDU, were less likely to achieve virologic suppression after ART initiation compared with male counterparts. This is in contrast to other evaluations that have found similar [12]–[14] or improved [11], [15], [16] virologic suppression compared to men. This analysis also demonstrates that women are at increased risk of virologic rebound after ART initiation. Similarly, previous studies have documented higher rates of virologic rebound among women [9], [11], [17], while other studies have not demonstrated this finding [12], [15].

Contradiction in the literature around gender-related differences in virologic responses to ART is likely in part due to heterogeneity in cohort demographics. In CANOC, a significantly higher proportion of women report a history of IDU (43.5% of women versus 28.8% of men, p<0.001), which reflects the composition of the Canadian HIV epidemic, particularly in British Columbia. More rapid HIV disease progression and poorer viro-immunological responses to ART among IDU have been previously reported in the literature [18]–[20]. This observation has been explained by suboptimal adherence [21] and treatment interruptions, with continued (active) drug use often contributing. In accordance with our findings, a recent North American cohort study demonstrated that individuals with a history of IDU were less likely to initiate ART or achieve virologic suppression [20]. Further, it is possible that in addition to IDU, female participants in CANOC have other psychosocial, structural, and other barriers to care not captured in this analysis such as competing circumstances that may include mental health and addiction issues, housing challenges, food insecurity, and other comorbid conditions [18].

When women assumed to be taking ART for prevention of vertical HIV transmission were removed from the analysis, there was a notable change in the overall virologic response to ART demonstrated by female participants. Gender-related differences in time to virologic rebound decreased but remained significant for participants without IDU history, however became insignificant among participants with IDU history. A previous publication exploring the influence of ART use for prevention of vertical HIV transmission on virologic response to ART demonstrated that pregnancy accounted for considerable gender-related variation in virologic rebound [11]. It is probable that ART discontinuation post-natally accounts somewhat for the gender-related difference in virologic rebound, however there are likely additional sociodemographic and psychosocial variables that influence adherence among women that are unaccounted for in this study.

Adjusting for presumed ART use in pregnancy accounted for the gender-related difference in time to virologic suppression observed in participants without IDU history, whereas the gender-related difference decreased but remained statistically significant among participants with a history of IDU. Findings from the aforementioned prior study demonstrated no change in gender-related difference in time to virologic suppression when adjusting for presumed ART use for prevention of vertical HIV transmission [11].

Gender differences in survival after ART initiation were not observed in this study, a finding that has also been reported in other settings [12], [22]. However, other studies have documented gender-related mortality differences [23]. A publication by the Johns Hopkins HIV Clinic Cohort demonstrated an increased rate of disease progression and mortality in women and IDU receiving ART compared with men [19], while a recent publication assessing patterns of HIV/AIDS mortality over time in Canada observed that standardized death rates were generally higher in men [24].

Readers of this work should consider several limitations. CANOC includes data from only three provinces, and a clinic-based selection bias exists, as included data from British Columbia includes the entire sample of people on ART province-wide while data from Ontario and Quebec come from a selection of clinics. In our analyses we did not consider antiretroviral adherence, an important predictor of virologic outcomes, as these data are not currently available. A further limitation is the potential for missing data, as by definition of our research question individuals in CANOC with missing IDU history information were removed from the study. However, when considering gender differences in clinical outcomes among persons in our cohort excluded from these analyses due to unknown IDU status (n = 2376, 14% female), increased risk of poorer outcomes among women remained evident for virologic suppression (AHR = 0.71, 95% CI = 0.62–0.82, p<0.001) and rebound (AHR = 2.66, 95% CI = 1.88–3.78, p<0.001) (data not shown). In this sub-sample women also had an increased mortality risk (AHR = 1.52, 95% CI = 1.06–2.18, p = 0.024) (data not shown).

We also acknowledge that identifying women taking ART for prevention of vertical HIV transmission by adopting the algorithm described presents margin for error. Lastly, between-cohort heterogeneity in methods used to ascertain mortality exist; mortality estimates here may thus be an underestimation of the true burden, particularly at contributing sites that do not link to vital statistics registries.

In conclusion, HIV-positive women in CANOC are at heightened risk for poor clinical outcomes. As women’s experiences of HIV infection and ART are unique, tailored services that respond to women’s needs are critical for improving health outcomes [25], [26]. The gender and risk associated differences in treatment outcomes demonstrated in this analysis likely reflect an interplay between structural, psychosocial, and biological factors. Further understanding of the intersections between gender and other factors augmenting risk is needed to identify those at risk for suboptimal therapeutic outcomes and maximize the benefits of ART in Canada.
